# Acceptance status and influencing factors of advance care planning among elderly patients with chronic disease

**DOI:** 10.1097/MD.0000000000040526

**Published:** 2024-12-06

**Authors:** Xiu Zheng, Yunlei Gao, Xin-Xin Yan, Xiaoxu Zhu, Chunqi Wang

**Affiliations:** a Department of Geriatric, Aerospace Center Hospital, Peking University Aerospace School of Clinical Medicine, Beijing, PR China.

**Keywords:** advance care planning, chronic diseases, elderly, palliative care

## Abstract

The aging population has become a severe public health issue both in China and globally. Over 180 million elderly people in China have chronic diseases, with 75% of them suffering from 2 or more chronic diseases, posing significant threats to their independence and quality of life, and imposing immense mental and disease burdens on patients and their families. So our research analyzes the influencing factors based on the current acceptance status of advance care planning (ACP) among elderly patients with chronic diseases. Using convenience sampling, 120 elderly inpatients from the geriatrics department of a tertiary hospital in Beijing, from June 2023 to March 2024, were selected. Surveys included a general information questionnaire, an ACP acceptance questionnaire, and the revised version of the Terminal Care Experience and Death Attitude Profile. The total ACP acceptance score among elderly patients with chronic diseases was 73.98 ± 9.84 points. Factors influencing ACP acceptance included fear of death, whether the patient had cared for a dying relative, duration of chronic illness, presence of complications, natural acceptance, and religious beliefs (*P* < .05). The acceptance level of ACP among elderly patients with chronic diseases is moderately high. Medical staff should provide more choices to enhance the life value of elderly patients with chronic diseases based on fully respecting their wishes.

## 1. Introduction

Population aging is a global phenomenon: virtually every country in the world is experiencing growth in the size and proportion of older persons in their population. There were 703 million persons aged 65 years or over in the world in 2019. The number of older persons is projected to double to 1.5 billion in 2050.^[1]^ According to a report by the China Development Foundation, by 2025, over 210 million people in China will be aged 65 and above, accounting for about 15% of the total population. By 2035, this figure will reach 310 million, representing 22.3% of the total population, indicating China has entered a stage of severe aging.^[2]^ Population aging will put increased financial pressure on old-age support systems: in countries where public transfers, population aging will increase the fiscal pressure on public transfer systems; in countries where public transfers are relatively low, individuals and families face greater pressure to finance their consumption during old-age.^[1]^

Noncommunicable diseases (NCDs), also known as chronic diseases, tend to be of long duration and are the result of a combination of genetic, physiological, environmental and behavioral factors. NCDs kill 41 million people each year, equivalent to 74% of all deaths globally. People of all age groups, regions and countries are affected by NCDs.^[3]^ The age structure of the population is closely related to the incidence of diseases. Elderly individuals have the highest prevalence and incidence rates of chronic diseases. Over 180 million elderly people in China have NCDs, with 75% of them suffering from 2 or more NCDs,^[3]^ posing significant threats to their independence and quality of life, and imposing immense mental and disease burdens on patients and their families.^[4]^

NCDs threaten progress towards the 2030 Agenda for Sustainable Development. The persistent treatment characteristics of chronic diseases, the escalating medical burden, and the rapidly increasing demand for medical services underscore the importance of palliative care. The WHO’s Global Action Plan for the Prevention and Control of NCDs 2013 to 2020 highlights that palliative care is an essential component of a comprehensive response to NCDs.^[5]^ WHO estimates that, some 20 million people need end-of-life palliative-care services each year, around 80% live in low-and middle-income countries; some 67% are elderly (>60 years of age), whereas about 6% are children.^[6]^ Palliative care can improve the quality of life of patients and of their families, but also benefits health care systems by reducing unnecessary admissions to hospital and use of health care services.^[7]^As a crucial part of palliative care, advance care planning (ACP) emphasizes the process where patients, while conscious, discuss and express their wishes for future medical care during critical illness with healthcare providers and/or family members in advance.^[8]^ Family members, as the primary caregivers of elderly patients with chronic diseases, play a significant role in establishing ACP.^[9]^ This study focused on the current acceptance status of ACP among elderly patients with chronic diseases and analyzed its influencing factors, providing scientific evidence for improving the quality of life of this population.

## 2. Subjects and methods

### 2.1. Study design and participants

Using convenience sampling, 120 elderly inpatients from the geriatrics department of a tertiary hospital in Beijing, from June 2023 to March 2024, were selected. Patients aged ≥ 60years or with at least one of the following characteristics were included: Clinically diagnosed with chronic diseases (including hypertension, diabetes, coronary heart disease, cerebral infarction, rheumatoid arthritis, asthma, etc); clear consciousness and able to cooperate; informed consent and voluntary participation in this study. Exclusion criteria were: combined with severe physical diseases or cognitive impairments that make it difficult to clearly express their thoughts.

Sample size: formal investigation sample size: according to Kendall criterion,^[10]^ the sample size is 5 to 10 times of the independent variable. There are 19 variables included in statistical analysis in this study, and 95 cases are calculated according to 5 times of the variable. Considering the sample loss rate of 10% to 20%, the sample size was finally determined to be 120 cases.

### 2.2. Research tools

General Information Questionnaire designed by the researchers, it includes sociodemographic factors such as age, gender, ethnicity, education level, marital status, preretirement occupation, living situation, income and expenditure, medical insurance, religious beliefs, etc; disease-related factors such as the type and number of chronic diseases, duration of illness, presence of complications, and daily activity ability. ACP Acceptance Questionnaire developed by Chinese scholar Ren Xiaojing et al,^[11]^ includes 3 dimensions: patients’ attitudes, feelings, and intentions regarding ACP, with a total of 19 items. The questionnaire is scored on a Likert 5-point scale, from “strongly disagree” to “strongly agree,” scored 1 to 5 respectively. The total score ranges from 19 to 95, with higher scores indicating higher acceptance of ACP. The total Cronbach α of the questionnaire was 0.869, and the Split-half reliability was 0.816. This scale is mainly used to survey patients’ acceptance of ACP and has good reliability and validity. The Previous End of Life Experiences Scale designed by Chinese scholar Wang Xinru^[12]^ referencing Hsiungtll’s Chinese version, consists of 6 questions. It asks patients about 6 aspects of medical experiences: whether they have suffered from fatal diseases or major accidents; whether relatives have suffered from fatal diseases or major accidents; whether they have been a patient in the ICU; whether relatives have been ICU patients; whether they have cared for dying relatives; whether they have participated in discussions on medical measures to prolong the life of terminally ill family members. Subjects select “yes” or “no” based on their actual situation, making it convenient for application to the population of chronic disease patients in China. The final questionnaire is death attitude profile-revised (DAP-R). The DAP-R scale was translated and revised by Chinese scholar Zhu Hailing,^[13]^ including 5 dimensions: natural acceptance, approach acceptance, escape acceptance, death fear, and death avoidance. Each dimension contains 5 items, scored on a Likert 5-point scale, from “strongly disagree” to “strongly agree,” scored 1 to 5, respectively. Higher scores indicate a stronger inclination towards that dimension of death attitude. This scale is widely used among the elderly and chronic disease patients, effectively measuring their death attitudes with good reliability and validity, with Cronbach α coefficient of each dimension ranging from 0.59 to 0.84.

### 2.3. Data collection

A database was established using Epidata 3.1, with data entered by 2 individuals for cross-checking. Data analysis was performed using SPSS 21.0. Descriptive statistics for quantitative data were expressed as mean ± standard deviation, and categorical data were described using frequency and percentage. Comparisons between groups were made using *t* tests or one-way ANOVA. Correlation analysis was performed using Pearson or Spearman correlation. Multiple linear stepwise regression analysis was used to analyze the influencing factors of ACP acceptance, with *P* < .05 indicating statistically significant differences.

## 3. Results

### 3.1. General information

A total of 120 elderly patients completed the questionnaire, including 47 males (39.2%) and 73 females (60.8%). 95 people (79.2%) had more than 2 chronic diseases. Seventy-two (60%) had a religious affiliation (Table 1). (The statistical results “*P* < .05” showed statistical difference.)

**Table 1:** General information and acceptance score.

Item	Category	Cases (n)	Percentage (%)	ACP acceptance score (Mean ± SD)	Test statistic value	*P*-value
Age (years)	60–69	40	33.3	75.45 ± 9.08	3.012†	.053
	70–79	54	45.0	74.85 ± 8.89		
	≥80	26	21.7	69.89 ± 11.90		
Gender	Male	47	39.2	72.21 ± 9.67	‐1.585*	.116
	Female	73	60.8	75.11 ± 9.84		
Ethnicity	Han	117	97.5	67.00 ± 11.27	‐1.247*	.215
	Minority	3	2.5	74.15 ± 9.79		
Education level	Primary or below	16	13.3	65.75 ± 9.36	15.862†	<.001
	Junior to senior	54	45.0	71.96 ± 9.80		
	University or above	50	41.7	78.78 ± 7.35		
Marital status	Married	97	80.8	74.27 ± 9.71	0.966†	.384
	Divorced or widowed	5	4.2	68.00 ± 7.52		
	Single	18	15.0	74.06 ± 10.94		
Residence	Rural	21	17.5	73.43 ± 10.05	‐0.279*	.781
	Urban	99	82.5	74.09 ± 9.84		
Income situation	Surplus	34	28.3	74.56 ± 11.23	0.164†	.849
	Balanced	80	66.7	73.86 ± 9.05		
	Deficit	6	5.0	72.17 ± 13.01		
Health insurance	None	6	5.0	79.33 ± 7.12	1.374*	.172
	Yes	114	95.0	73.69 ± 9.90		
Religious belief	None	48	40.0	69.29 ± 9.72	‐4.607*	<.001
	Yes	72	60.0	77.80 ± 8.66		
Number of chronic diseases	1	25	20.8	63.24 ± 8.34	33.269†	<.001
	2	47	39.2	74.40 ± 7.17		
	≥3	48	40.0	79.15 ± 8.39		
Duration of chronic disease (years)	<3	55	45.8	71.33 ± 9.95	3.985†	.021
	3–10	52	43.4	75.92 ± 8.87		
	>10	13	10.8	77.39 ± 10.90		
Presence of complication	Yes	31	25.8	72.01 ± 9.89	‐3.924*	<.001
	No	89	74.2	79.61 ± 7.25		
Daily living ability score	Independent	38	31.7	73.21 ± 9.67	0.409†	.747
	Mild dependence	47	39.2	73.45 ± 9.43		
	Moderate dependence	22	18.3	75.32 ± 9.88		
	Severe dependence	13	10.8	75.85 ± 12.21		

* *t* value.

† *F* value.

The average ACP acceptance score of the elderly patients with chronic diseases was 73.98 ± 9.84 points (ranging from 46–87 points) (Table 2).

**Table 2:** ACP acceptance scores among elderly patients with chronic diseases.

Item	Score range	Score (mean ± SD)	Item mean score (mean ± SD)
ACP attitude	7–15	11.86 ± 2.32	3.95 ± 0.77
ACP feelings	24–50	42.10 ± 6.43	3.83 ± 0.58
ACP intentions	8–25	20.02 ± 3.82	4.00 ± 0.76
Total ACP acceptance score	46–87	73.98 ± 9.84	3.89 ± 0.52

### 3.2. Distribution of each dimension

Table 3 shows among the end-stage related medical experience of elderly patients with chronic diseases, 81 (67.5%) had experienced. “Have I suffered fatal diseases or major accidents”; “Has a loved one suffered a fatal illness or major accident?” 61 (50.8%) had 71 (59.2%) had been patients in the ICU; 70 patients (58.3%) had relatives who had been in intensive care unit; 51 people (42.5%) have taken care of their relatives who are close to death. Sixty-seven people (55.8%) participated in the discussion on what medical measures should be taken to keep their family members alive. (The statistical results “*P* < .05” showed statistical difference.)

**Table 3:** End-of-life medical experience among elderly patients with chronic diseases.

Item	Cases (n)		Percentage (%)	ACP acceptance score (mean ± SD)	Test statistic value	*P*-value
1. Have you ever suffered from a fatal disease or major accident?	Yes	81	67.5%	74.79 ± 9.82	‐1.312	.192
	No	39	32.5%	72.28 ± 9.77		
2. Has a relative ever suffered from a fatal disease or major accident?	Yes	61	50.8%	73.62 ± 10.13	0.397	.692
	No	59	49.2%	74.34 ± 9.59		
3. Have you ever been an ICU patient?	Yes	71	59.2%	74.70 ± 10.24	-0.978	.330
	No	49	40.8%	72.92 ± 9.21		
4. Has a relative ever been an ICU patient?	Yes	70	58.3%	73.91 ± 10.08	0.080	.937
	No	50	41.7%	74.06 ± 9.59		
5. Have you ever cared for a dying relative?	Yes	51	42.5%	79.37 ± 7.60	‐6.035	<.001
	No	69	57.5%	69.97 ± 9.43		
6. Have you ever participated in discussions about medical measures to prolong the life of a terminally ill family member?	Yes	67	55.8%	75.18 ± 9.62	-1.516	.132
	No	53	44.2%	72.45 ± 9.98		

Table 4 shows in the correlation analysis of ACP acceptance and death attitude in elderly patients with chronic diseases, the realistic death fear and death avoidance were negatively correlated with the acceptance of advance medical treatment in elderly patients with chronic diseases, while the natural acceptance was positively correlated with the acceptance of advance medical treatment in elderly patients with chronic diseases. The results show that the elderly are more willing to participate in ACP discussion if they adopt a positive attitude towards death. (The statistical results “*P* < .05” showed statistical difference.)

**Table 4:** Correlation analysis between ACP acceptance and death attitudes among elderly patients with chronic diseases.

Item	ACP attitude	ACP feelings	ACP intentions	ACP acceptance
Death fear	‐0.310***	‐0.309***	‐0.259**	-0.375***
Death avoidance	‐0.231*	‐0.367***	‐0.260**	-0.395***
Natural acceptance	0.283**	0.492***	0.359***	0.527***
Approach acceptance	0.103	0.118	0.193*	0.176
Escape acceptance	‐0.079	‐0.281	‐0.028	‐0.213*

* *P* < .05.

** *P* < .01.

*** *P* < .001.

Table 5 shows results of multiple linear stepwise regression analysis on influencing factors of ACP acceptance among elderly patients with chronic diseases.

**Table 5:** Results of multiple linear stepwise regression analysis on influencing factors of ACP acceptance among elderly patients with chronic diseases.

Variable	Regression coefficient	Standard error	Standardized regression coefficient	*t* value	*P*-value
Constant	60.209	6.059	–	9.937	<.001
Religious belief	5.523	1.189	0.276	4.644	<.001
Duration of chronic illness	4.016	0.923	0.273	4.350	<.001
Presence of complications	4.383	1.540	0.196	2.847	.005
Have you ever taken care of a dying relative?	7.214	1.243	0.364	5.802	<.001
Natural acceptance	3.476	0.855	0.315	4.063	<.001
Death fear	-2.446	0.706	-0.222	-3.464	.001

*Note*: *R* = 0.827, *R*² = 0.687, adjusted *R*² = 0.655, *F* = 23.574, *P* < .001.

Using ACP acceptance score as the dependent variable and factors with statistical significance from univariate analysis as independent variables, multiple linear stepwise regression analysis was performed. Variable assignments were as follows: Education level, primary or below = 1, junior to senior high = 2, high school and above = 3; Religious belief, none = 0, yes = 1; Number of chronic diseases, 1 = 1, 2 = 2, ≥3 = 3; Duration of chronic illness (years), <3 = 1, 3–10 = 2, >10 = 3; Presence of complications, yes = 1, no = 2; Whether the patient has cared for a dying relative, yes = 1, no = 2; Scores for death fear, death avoidance, natural acceptance, approach acceptance, and escape acceptance dimensions in the DAP-R were entered as raw values. The results showed that death fear, whether the patient has cared for a dying relative, duration of chronic illness, presence of complications, natural acceptance, and religious belief were influencing factors of ACP acceptance among elderly patients with chronic diseases, as shown in Table 5.

## 4. Discussion

### 4.1. The acceptance level of ACP among elderly patients with chronic diseases is above average

In this study, the overall score for the acceptance of ACP among elderly patients with chronic diseases was 73.98 ± 9.84, which is above average. This score is higher than that of elderly patients with chronic diseases in nursing homes^[14]^ and middle-aged cancer patients,^[15]^ indicating the ongoing development and promotion of ACP in China. The reasons for this may include the increasing aging population, the protracted treatment characteristics of chronic diseases, the heavy burden on medical resources, and the development of palliative care, which have all contributed to the advancement of ACP. Initially, this concept started with personal self-initiated websites and has now progressed to being recognized in specific areas through local legislation, such as the “Shenzhen Special Economic Zone Medical Regulations,” which affirm the validity of advance directives in certain regions.^[16]^ This represents an innovative measure for patient autonomy at the end of life. However, compared to other regions and China’s Hong Kong and Taiwan, the development and practice of ACP remain slow and lagging behind. Many people still have a blank understanding of ACP.^[17]^ Therefore, as healthcare professionals, we should respect patients’ wishes and provide more options for elderly patients with chronic diseases to realize the value of life.

### 4.2. The acceptance of ACP among elderly patients with chronic diseases is influenced by various factors

#### 4.2.1. Patients with religious beliefs, longer duration of chronic illness, and complications are more likely to accept ACP

This study shows that elderly patients with chronic diseases who have religious beliefs are more likely to accept ACP, which is consistent with the findings of Zhu T et al^[18]^ and Zhao Yufei.^[19]^ Religious belief, as a complex social and cultural phenomenon, has a widespread and profound impact on the elderly population. Due to declining physical functions, the torment of recurring diseases, and the significant threat of death, elderly patients with chronic diseases experience a tremendous impact on their health and psychology. Religious beliefs can enhance the subjective well-being of elderly patients to some extent, enabling them to have a more positive view of life and death.^[20]^ Therefore, in the growing population of elderly people with religious beliefs, religion, as an important carrier of human culture, should be viewed with a more objective and rational attitude to fully utilize its positive role.

The study also shows that elderly patients with chronic diseases of longer duration and those with complications are more likely to accept ACP, which is consistent with the findings of Zhang Kun et al.^[21]^ According to reports, the probability of elderly people suffering from chronic diseases is 4.2 times higher than that of the general population,^[22]^ and deaths caused by chronic diseases account for 88.5% of total deaths, with medical expenses due to chronic diseases exceeding 70% of total disease-related medical expenses.^[23]^ The difficulty and chronicity of treating chronic diseases not only negatively impact the physical functions, quality of life, and mental health of the elderly but also significantly increase the economic and caregiving burdens on families. Additionally, as the condition recurs and the disease course extends, patients’ understanding of their illness gradually increases, leading to more rational predictions about their treatment and prognosis. Consequently, they are more willing to seek more reasonable treatment options. Therefore, elderly patients with chronic diseases of longer duration and those with complications have a higher acceptance of ACP, which also provides more choices for their disease treatment.

#### 4.2.2. Elderly patients with chronic diseases who have personally cared for dying relatives are more likely to accept ACP

The results of this study show that elderly patients with chronic diseases who have personally cared for dying relatives are more likely to accept ACP, consistent with the findings of Rami et al.^[24]^ The experience of having a family member in the intensive care unit can cause significant psychological and social stress for both patients and family members, such as shock, denial, guilt, and fear of losing a loved one.^[24]^ When facing the process of a loved one’s death, individuals are often prompted to reflect on their own end-of-life choices. Thus, having personally cared for dying relatives can increase acceptance of ACP. On the other hand, with the increasing visibility of palliative care, elderly patients with chronic diseases facing long-term illness or witnessing the suffering of their family members can consider new options. Palliative care, as an essential aspect of addressing population aging and social healthcare needs, is suitable for any stage of life-threatening diseases, including chronic diseases. Considering palliative care in the early stages of illness can improve quality of life, reduce unnecessary hospitalizations, and promote the rational use of medical resources.^[25]^

#### 4.2.3. Fear of death and death avoidance negatively correlate with acceptance of ACP among elderly patients with chronic diseases, while natural acceptance positively correlates

The results of this study indicate that fear of death and death avoidance among elderly patients with chronic diseases negatively correlate with their acceptance of ACP, consistent with the findings of Piers et al.^[26]^ Elderly patients who adopt an avoidance attitude towards death are less likely to participate in discussions about ACP, suggesting that a negative attitude towards death is an obstructive factor for accepting ACP. Conversely, a natural acceptance attitude towards death positively correlates with the acceptance of ACP among elderly patients with chronic diseases. This indicates that elderly patients who naturally accept death are more likely to accept ACP. Elderly patients with a correct attitude towards death can calmly accept it and approach ACP with a positive and optimistic mindset, providing them with more options throughout different stages of life and the possibility of dying with dignity. Therefore, as healthcare professionals and advocates and practitioners of palliative care, it is essential to strengthen death education and guide elderly patients with chronic diseases to establish correct death concepts, thereby improving their acceptance and practice of ACP.

## 5. Conclusion

The results of this study show that the acceptance of preestablished medical care for elderly patients with chronic diseases is at a moderate to high level, indicating the rapid development of palliative care in China. However, compared to other regions such as Hong Kong and Taiwan, the development of preestablished medical care in mainland China is still slow and lagging behind. Countries such as the United States, Canada, and Australia not only have ACP related laws, but have also achieved significant results in the medical field, including malignant tumors, cardiovascular diseases, and respiratory diseases; however, influenced by Confucianism and traditional Chinese culture, the protection of the rights and legal system for ACP patients in mainland China is still slow. So as healthcare professionals, disseminators and practitioners of palliative care, we actively promote the development of palliative care in China. We will provide more choices for elderly chronic disease patients in need to realize their life value on the basis of fully respecting their wishes. However, this study also has certain limitations. The selection of our research subjects only included patients admitted to our geriatric department, which limits the sample size. Therefore, in our next research, we can consider expanding the sample size and conducting a multicenter study; it is also possible to explore the acceptance of ACP among different patient groups in different medical environments; at the same time, as important decision-makers in preestablished medical care, the acceptance of preestablished medical care by patient family members can also be considered as the next research direction.

## Author contributions

**Data curation:** Xiu Zheng.

**Formal analysis:** Xin-Xin Yan.

**Investigation:** Xiaoxu Zhu, Chunqi Wang.

**Resources:** Yunlei Gao.

**Writing – original draft:** Xiu Zheng.
